# Supplementary figures and images for: miR-203 secreted in extracellular vesicles mediates the communication between neural crest and placode cells required for trigeminal ganglia formation
Source: PLoS Biol. 2024 Jul 22;22(7):e3002074. doi: 10.1371/journal.pbio.3002074 (PMC11293684; doi:10.1371/journal.pbio.3002074)

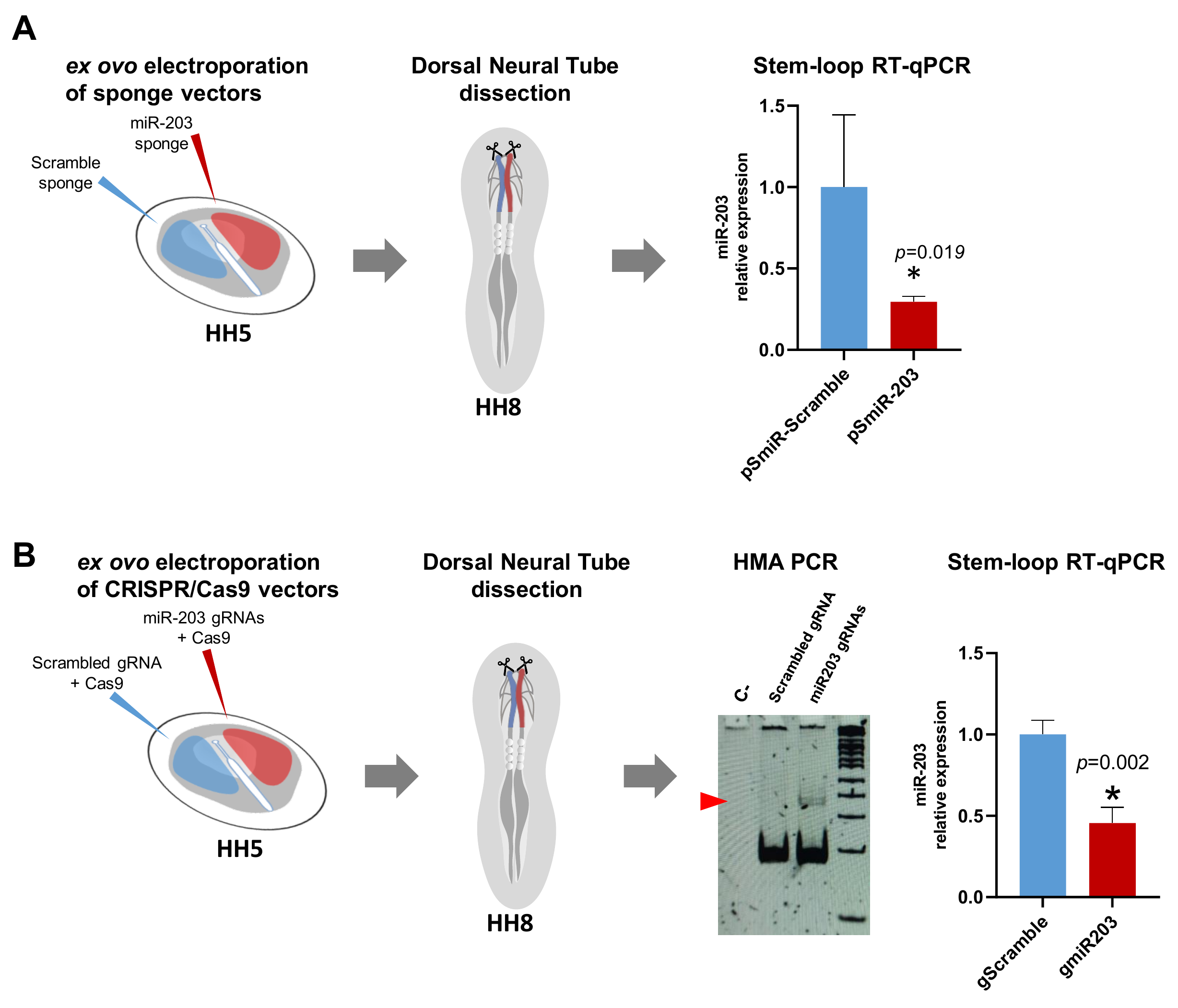

Supplement: S1 Fig — Stem-loop RT-qPCR confirm sponge-mediated (A) or CRISPR/Cas9 (B) loss of miR-203 on the treated side compared with the contralateral side of the same group of embryos. Asterisk (*) indicate significant differences by Student’s t test. Values are means (A, n = 4; B, n = 3) ± SD. Heteroduplex mobility assay PCR (HMA PCR) showed multiple heteroduplex bands from miR-203 gRNA-treated embryos (red arrowhead) and a single band obtained in scrambled gRNA-electroporated embryos. (TIFF) [file pbio.3002074.s001.tiff]

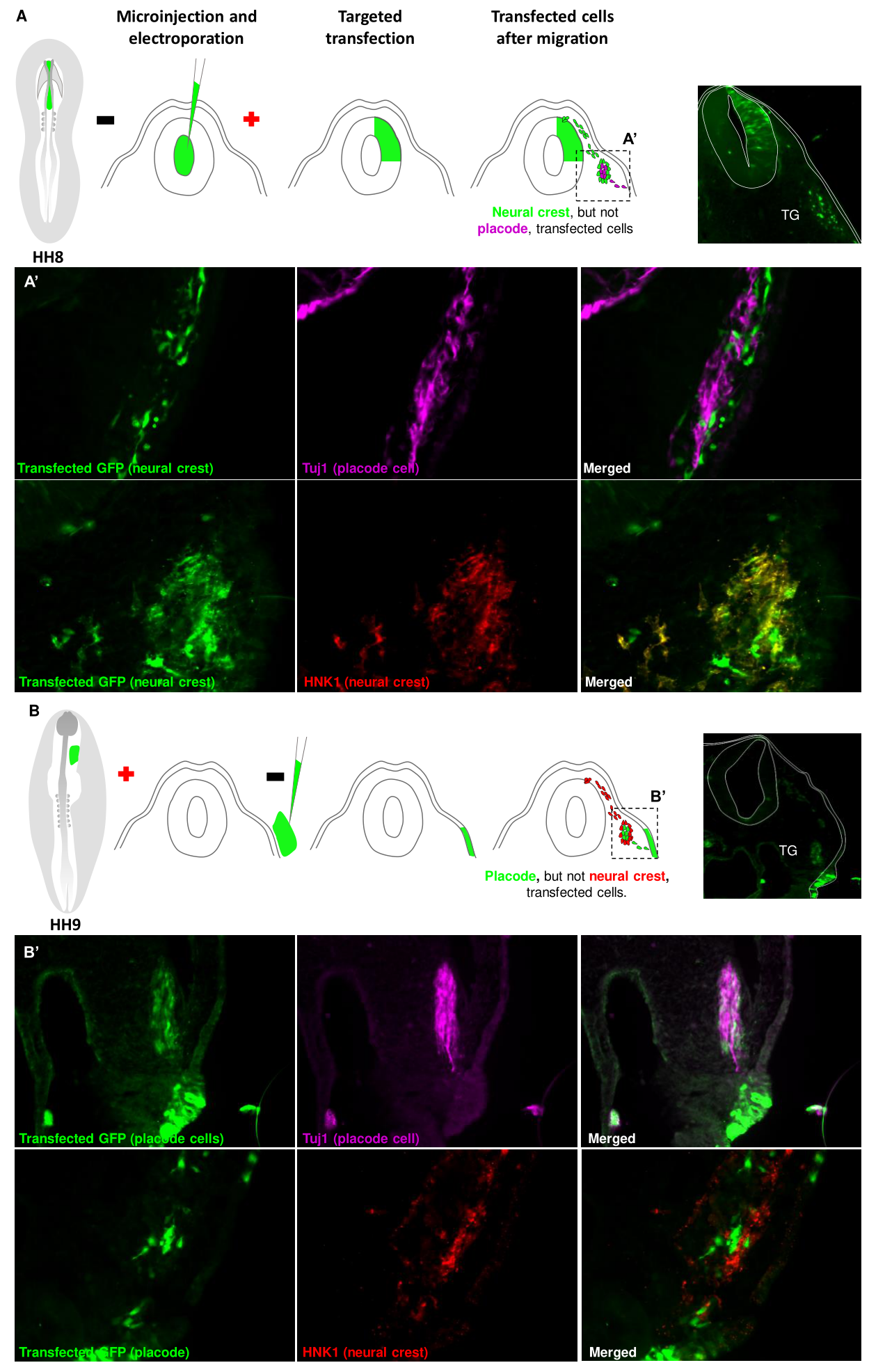

Supplement: S2 Fig — (A) Representation of HH8 stage chick embryos and transverse views of microinjection into the lumen of the NT, position of the electrodes for electroporation, targeted transfection and transfected neural crest cells, but not placodal cells, after migration. (A’) Shows transfected GFP neural crest cells co-localizing with HNK1, but not Tuj1, marker. (B) Representation of HH9 stage chick embryos and transverse views of microinjection on top of the ectoderm region, position of the electrodes for electroporation, targeted transfection and transfected placodal cells, but not neural crest after migration. (B’) Shows transfected GFP placodal cells co-localizing with Tuj1, but not HNK1, marker. (TIFF) [file pbio.3002074.s002.tiff]

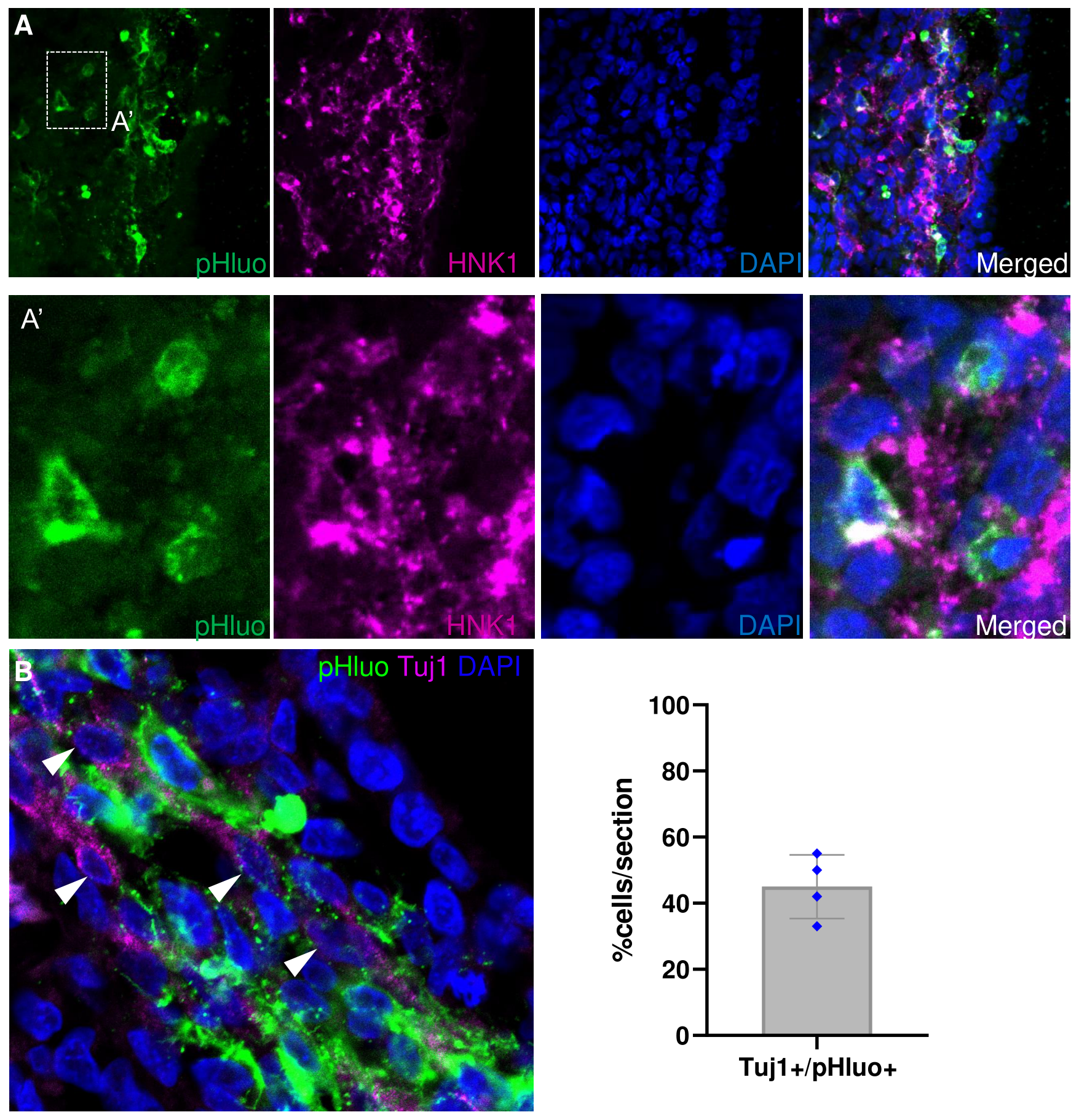

Supplement: S3 Fig — (A) Transverse section of HH12 embryos sowing migratory cells electroporated with pHluo and immunostained for HNK1 (magenta). (A’) Zoom of box in A shows all migratory pHluo+ cells co-localized with HNK1 marker. (B) Transverse section through the TG showing NC cells electroporated with pHluo and placode cells immunostained for Tuj1 (magenta). Bar graph shows the percentage of Tuj1+ cells that has pHluo puncta incorporated into their cytoplasm (white arrowheads exemplified Tuj1+/pHluo+ cells) per section (3 to 4 sections have been observed) analyzed in 4 different embryos (see S1 Data). Values are means ± SD. (TIFF) [file pbio.3002074.s003.tiff]

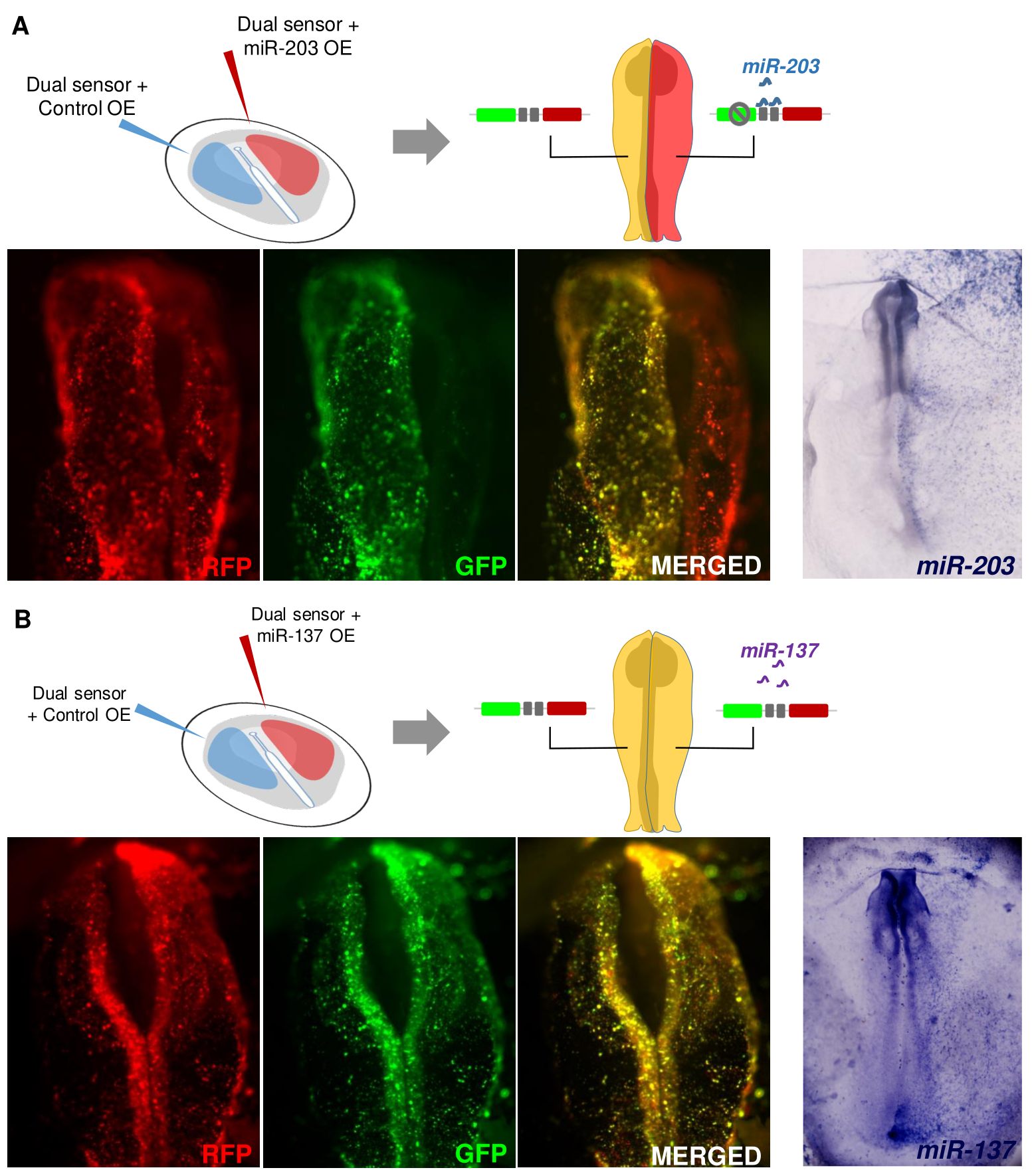

Supplement: S4 Fig — (A) We injected in the right side the miR-203 overexpressing vector (miR-203 OE) together with the dual-sensor vector (Dual sensor) containing 2 copies of complementary sequences to the mature miR-203 in the 3′ UTR of d4EGFPN, but not in the mRFPN. The surviving embryos showed a drastic reduction in the GFP signal of the sensor in the right side, where miR-203 is overexpressed, compared with the left control side where both GFP and RFP are clearly detected. Successfully overexpress a mature miR-203 was evidenced by in situ hybridization using LNA probes. (B) Control embryos were injected in the right side the miR-137 overexpressing vector (miR-137 OE) together with the Dual sensor vector containing 2 copies of complementary sequences to the mature miR-203 in the 3′ UTR of d4EGFPN, but not in the mRFPN. The surviving embryos showed in both sides similar EGFP and GFP intensities. Successfully overexpress a mature miR-137 was evidenced by in situ hybridization using LNA probes. (TIFF) [file pbio.3002074.s004.tiff]

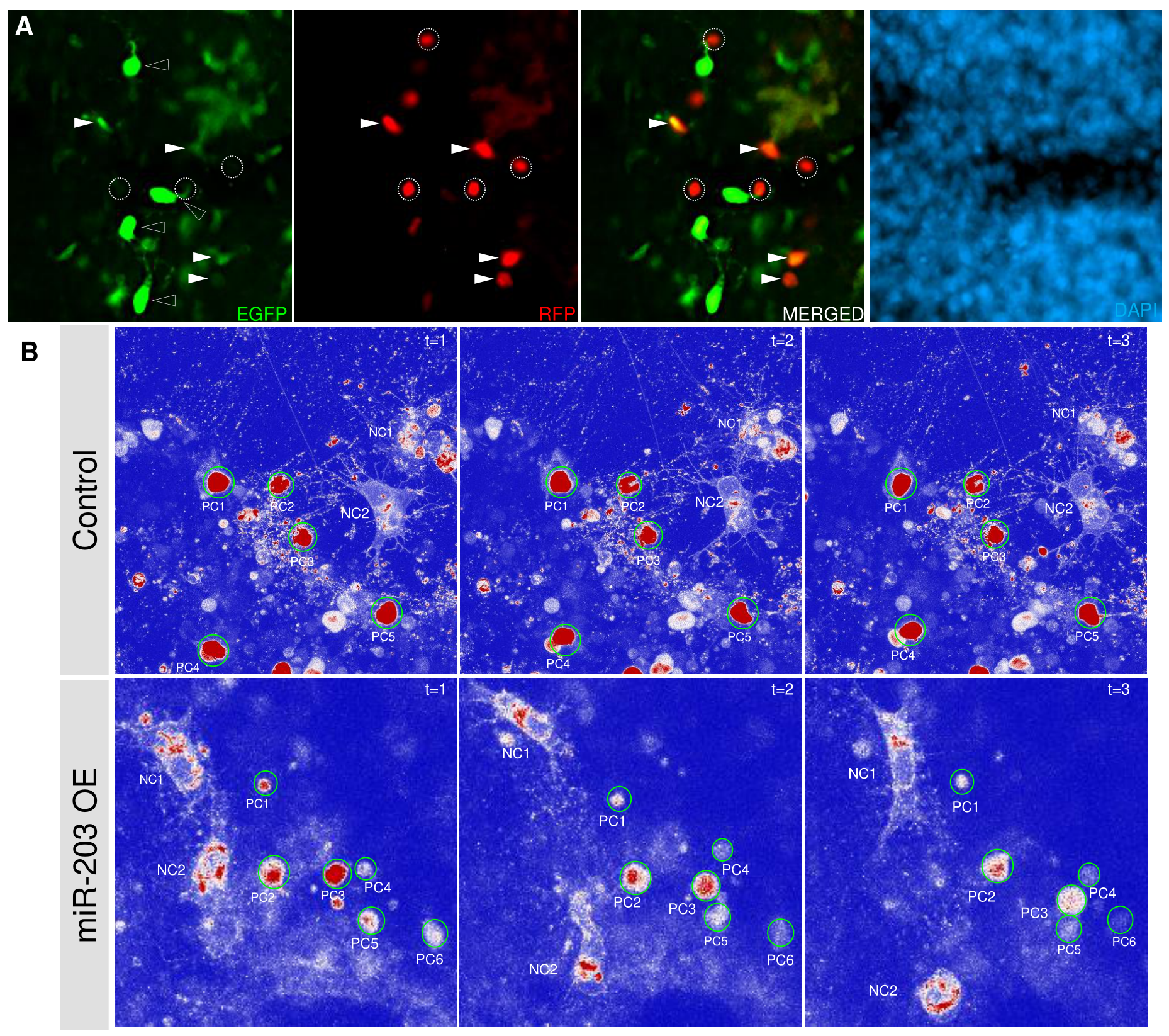

Supplement: S5 Fig — (A) Transverse section of HH17 embryos to identify migratory NC cells with cytoplasmic EGFP+ (black arrowhead), placode cells with nuclear EGFP+/RFP+ (white arrowhead) or only RFP+ (dotted circles), and DAPI staining to visualize the nuclei. (B) Time-lapse of co-cultured placode ectodermal cells (electroporated with the dual-colored sensor vector) and dorsal NT (electroporated with miR-203 OE or Control empty vectors) explants. Placodal cells (PC) interact with NC cells (NC) where the EGFP channel was pseudocolored (red>white>blue) to visualize the EGFP decay in placode cells over time. In control explants PC1-5 maintain high levels of EGFP fluorescence over the time, compared with the decay observe in PC1-6 in miR-203 OE explants. (TIFF) [file pbio.3002074.s005.tiff]

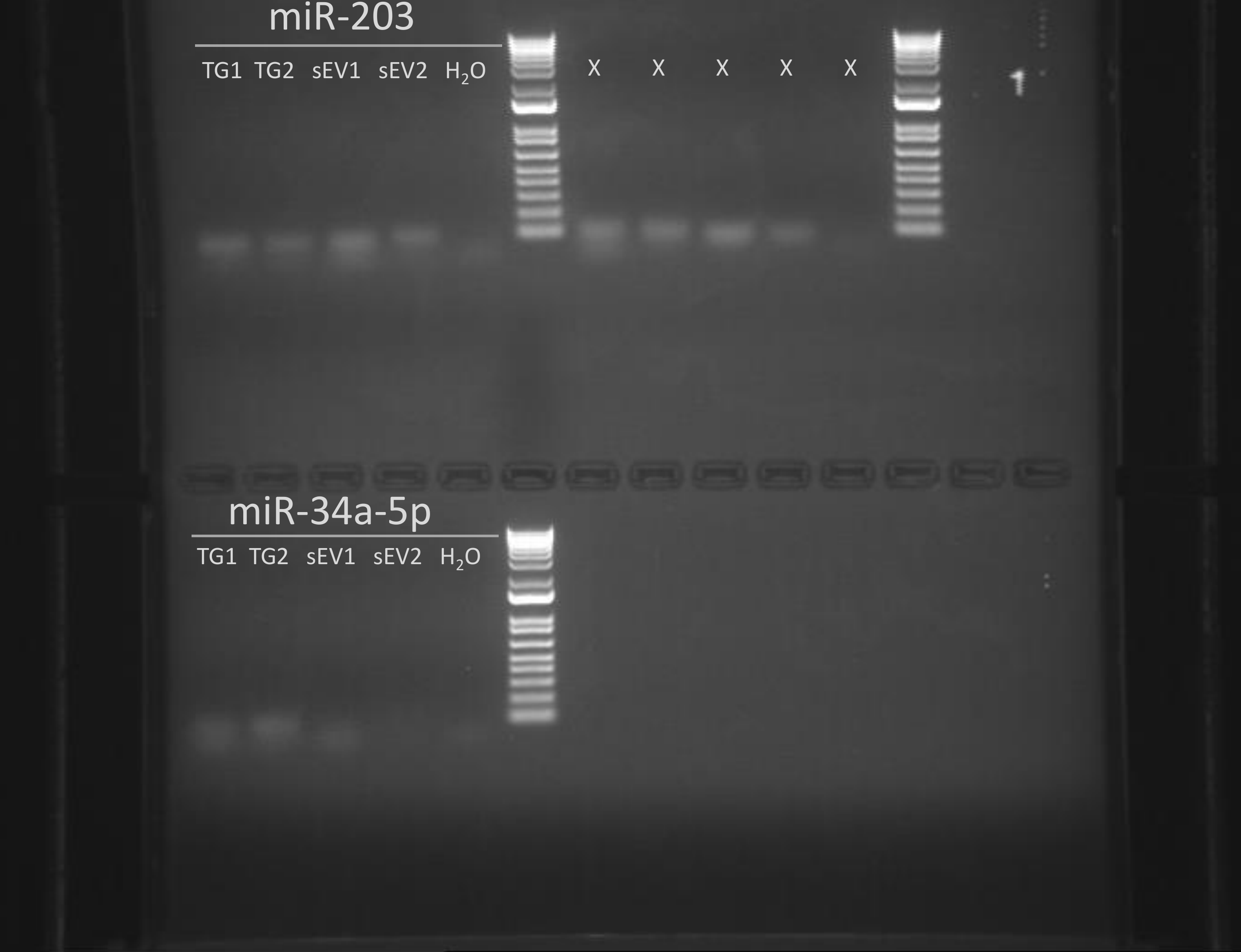

Supplement: S1 Raw Images — (TIFF) [file pbio.3002074.s014.tiff]
