# Supplementary material for: miR-203 secreted in extracellular vesicles mediates the communication between neural crest and placode cells required for trigeminal ganglia formation
Source: PLoS Biol. 2024 Jul 22;22(7):e3002074. doi: 10.1371/journal.pbio.3002074 (PMC11293684; doi:10.1371/journal.pbio.3002074)
Supplement: Attachment — Submitted filename: REVIEWS.pdf [file pbio.3002074.s015.pdf]

Response to REVIEWERS:

Reviewer #1:

This study has provided novel insights and revealed new mechanisms whereby the development of the trigeminal nucleus is regulated. However, there are several errors in the figures as well as inadequate explanation provided for certain observations. The detailed review is provided below.

We wish to convey our gratitude for the positive and constructive comments received. In response, we have made efforts to address the concerns raised by the reviewer in the points outlined below.

Detailed comments:

Fig.1:

1. In Fig. 1B, miR-203 is described as being absent from migrating NC cells but high magnification images are necessary for this to be convincing. Immunostaining with a marker for placode cells should also be used along with in situ hybridization for miR-203 to show whether miR-203 is expression is restored in the placode cells or the NC cells or both.

According to the reviewer's suggestion, we have now included higher magnification images of immunostaining with Tuj1, HNK1, and miR203. As can be observed, miR-203 is detected in both NC and placode cells at the coalescence (HH16) stage. At the condensation stage (HH20), miR-203 is mostly detected at the core of the ganglia where placode cells are mostly detected (Tuj1+).

2. Scale bar is missing in all figure panels of Figure 1.

Scale bars have been incorporated.

Fig. 2:

1. Authors have given reference to their previous paper (Sánchez-Vásquez et al., 2019) for the strategy of electroporation with miR-203 gain of function and loss of function construct where pCAG-GFP was used along with the desired construct for electroporation. The authors have not provided any information or data for the visualization of electroporated cells in fig 2A'. Either an electroporation marker or in situ for miR-203 is necessary to determine if miR203 overexpressing cells are the ones that have led to the ectopic condensation.

We thank the reviewer for pointing this out. We have to mention that most of the endogenous fluorescence is quenched after the ISH protocol. So, we initially visualized the embryos and photographed them before the ISH to assess for proper electroporation. Those images have been included as insets in Figure 2A. Sometime after the whole mount ISH and embryo sectioning, we could still see a weak GFP signal by overexposing the sections, but this makes a lot of noise. We have now included an inset image in Figure 2A' showing the GFP channel for the corresponding section.

We acknowledged that our electroporation system has been a matter of concern for most of the reviewers in this manuscript and would like to clarify that this is one of the most powerful tools we have in chick embryology and it is widely used in our field. Numerous publications have demonstrated that when the neural tube is transfected by electroporation most of the delaminating neural crest, but none of the placode cells that have a different embryonic origin, will be affected in only one side of the embryos. In a similar way, we could specifically target one side of placode cells without affecting the NCC population. We have now included a better scheme of our injection and electroporation setups **(Fig. S2A-B)**. Additionally, we presented results demonstrating that transfection of neural crest with a GFP construct results in no co-localization with Tuj1 (placode marker) but clearly co-localize with HNK1 (neural crest marker) **(Fig. S2A'**). Conversely, when placode cells are transfected with a GFP construct, there is no co-localization with HNK1, but they do co-localize with Tuj1 marker **(Fig. S2B'**). We hope that this experiment clarifies the specificity and robustness of our electroporation system.

For a comprehensive and detailed description of the methodology for in ovo electroporation in chick embryos to target specific tissues during early embryogenesis, we refer you to Chapter 6 of the book "Neural Crest Cells: Methods and Protocols" authored by McLennan and Kulesa, which is part of the

"Methods in Molecular Biology" series (Volume 1976). You can access the full chapter at this DOI link: https://doi.org/10.1007/978-1-4939-9412-0_6.

2. Authors have used HNK1 in Fig.1 and Sox10 in Fig.2 as markers for neural crest cells. The authors should provide a rationale for using two different markers to detect the NC cells.

Thank you for bringing this to our attention. In Figure 1, we opted to utilize the HNK1 antibody because we aimed to co-localize it with miR-203 expression detected via ISH. We selected this particular antibody due to its robust performance following the ISH protocol, as not all antibodies are equally effective following the methanol washes required during the ISH procedure.

In Figure 2, our choice to employ ISH was driven by our need to process large batches of embryos to assess the neural crest cell's ability to form the trigeminal ganglia. This decision was primarily dictated by our experimental requirements. It's worth noting that both HNK1 and Sox10 can serve as reliable neural crest cell markers.

3. Scale bars are missing in all figure panels.

Scale bars have been incorporated.

Fig. 3

1. Fig. 3B shows the effect of the loss of function of miR-203 in placodal cells where endogenous expression of miR-203 is absent according to the information given in the introduction. However, it has not been clearly demonstrated which cells (NC or placodal cells) express miR-203.

The reviewer brought up a pertinent concern. Our findings indicate that miR-203 is initially expressed in the neural crest (NC), with subsequent predominant detection occurring at the central region of the trigeminal ganglia, where the majority of placode cells are concentrated. Regrettably, we currently lack the necessary tools to selectively sort each cell type for a more precise quantification using RT-qPCR.

2. Authors claim that loss of miR-203 in neural crest cells does not lead to any defect in trigeminal ganglion condensation. However, previous work published by the same group (Sánchez-Vásquez et al., 2019) demonstrated early delamination of neural crest cells. An explanation needs to be provided as to why this early delamination was not observed in this case.

The reviewer has pointed out a very interesting observation that we have taken in consideration when our experiments were designed. In our previous publication, to affect early steps of NC delamination we used *ex ovo* electroporation to introduce our sponge vector at the gastrula stage (HH5). In this new work, we used *in ovo* electroporation at ~HH9 embryos (5 somites), which is ~2 h before the NC starts to delaminate (6 somites). It is well documented that the promoter contained in our plasmids (chicken b-actin) usually takes ~4-5 h to be transcribed, so we will start to have sponge transcripts way after the NC cells delaminate from the neural tube. This is the reason why we are not affecting the delamination by using this experimental design.

3. Marker for electroporation e.g. GFP expression from a co-electroporated construct or the same construct through IRES-GFP is needed to visualize manipulated cells in all experiments.

Unfortunately, as we mentioned before, the GFP fluorescence is mostly quenched after ISH. We have now included micrographs from the treated side (see insets) for the same embryos before the ISH processing.

4. Scale bar is missing from all the panels in this figure.

Scale bars have been included.

Fig. 4:

1. A detailed explanation of the experiment should be provided.

Figure legends have been modified for a better understanding.

2. In Fig. 4F, to be able to see if neural crest cells electroporated with pHluo-CD63-mScarlet are indeed releasing pHluo-positive sEVs, a marker for neural crest cells like Sox10 along with mScarlet+ and GFP+ fluorescence should be shown in transverse sections. Both mScarlet and pHluo are not clearly overlapping with DAPI staining.

We have now included a new supplementary **figure S3A-A'** showing electroporated neural crest cell with pHluo and immunostained with HNK-1 (neural crest marker) in transversal section. DAPI staining was also included to identify nucleus, but we have to clarify that both HNK-1 and pHluo staining primarily target the cytoplasm.

3. For Fig. 4G quantification is required to see how many Tuj1+ placode cells are positive for pHluo fluorescence.

Thank you for your suggestion. We have now incorporated a graph into supplementary Figure S3B, displaying the percentage of Tuj1+ cells that have incorporated pHluo puncta into their cytoplasm. We conducted this analysis on 3-4 sections from each of the 4 individual embryos. Our findings reveal that 45% of the placodal cells, as indicated by Tuj1 immunostaining, exhibited GFP+ puncta incorporation.

Fig. 5:

1. Detailed information on constructs used in this experiment must be provided.

Thanks for pointing this out. We have revised the M&M section, incorporating comprehensive details in the "DNA construct and electroporation" section.

2. Authors have electroporated Neural crest cells with pHluo and co-cultured them with placode cells. Since pHluo fluorescence can only be seen after the vesicle containing pHluo has been secreted outside. Therefore, it is not clear how authors are visualizing the NC cells that have secreted the vesicles containing pHluo.

The pHluo vector enables us to visualize both the EVs that are about to be released (resulting from multivesicular body [MVB] fusion with the extracellular membrane) and those that have already been released. Given that MVB formation and EV release represent a continuous process, we can consistently observe robust pHluo fluorescence within the neural crest cytoplasm throughout the entire time-lapse.

3. In Figure 5D, white arrowheads dictate intra-cytoplasmic vesicular structures containing pHluo+ puncta in placode cells. Since pHluo is pH sensitive hence it should not be fluorescent after being endocytosed by placode cells. An explanation for this needs to be provided.

This is indeed a compelling observation. The mechanism by which EVs are endocytosed by placode cells remains elusive. It's important to note that not all methods of endocytosis necessarily involve acidic compartmentalization that could trigger the pHluo signal to diminish. Additionally, it's conceivable that the duration of our time-lapse recording may not be adequate to capture the pHluo protein's attenuation within the placode cells. We have speculated in the discussion some possible mechanisms of how EVs can be endocytosed.

4. Scale bar is missing from all images of this figure.

Scale bars have been included.

Fig. 6:

1. In Fig 6A the schematic showing the miR-203 dual sensor, has target sequences for miR-203 incorrectly labeled as sponge sequences.

Thanks for pointing this out. Change have been made accordingly.

2. DAPI is absent from Fig.6B and B.'

We have included the DAPI staining as a supplementary figure S5.

3. Controls for this experiment such as electroporation with a dual sensor with scrambled target sequences for miR-203 and a mutated miR-203 that is incapable of binding to its target have not been carried out.

Our primary aim here is to illustrate the capacity of miR203 produced in neural crest cells to reach and exert an influence on placode cells. Although we agree with the reviewer that including all these controls would have been important, we avoided some of these due to the technical difficulties of the experiment presented here. We would like to highlight that the results showed in Figure 6 poses an important technical challenge as it involves two electroporations, and limited survival rate of embryos. Based on this, we considered that including all of these controls are unnecessary in this context. We

have overexpressed an empty vector in neural crest cells to demonstrate that the decline in GFP fluorescence is not attributable to factors inherent to the placode cells.
Alternatively, we have conducted additional experiments to demonstrate the specificity of our sensor vector (now incorporated as **supplementary Figure S4)**. In these experiments, *ex-ovo* electroporation enabled us to target the right side of the embryos with the dual sensor + miR-203 overexpressing (miR-203 OE) vectors and the left side of the same embryos with the dual sensor + control empty pCIG vector (Control OE). Co-electroporation of the dual sensor vector and the empty pCIG vector (left side) resulted in the majority of cells appearing yellow due to the expression of both GFP and RFP reporters **(Fig. S4A)**. In contrast, co-electroporation of the dual sensor vector and miR-203 OE vector (right side) led to the majority of cells being only red, indicative of the strong repression of the GFP reporter. In contrast, when we overexpressed another miRNA, such as miR-137, it failed to bind to the two complementary miR-203 sequences present in the dual sensor vector, and both RFP and GFP were visible on both sides of the embryos **(Fig. S4B)**. In both cases, we presented in situ hybridization results, utilizing specific LNA-probes, confirming effective overexpression of miR-203 and miR-137 on the right side of the embryos.

4. In panel Fig. 6B' the placode cells circled with dashed lines are incorrectly labeled as being GFP+/RFP-, rather they should be GFP-/RFP+.

Sorry for this typo and thanks for pointing it out. Changes have been accordingly.

5. Image in Fig.6B' does not have a clear distinction between neural crest cells and placode cells as both express GFP. Therefore, a marker for neural crest cells shown along with EGFP would be useful.

Regrettably, our microscope is currently equipped with a limited number of available channels for neural crest detection. In this particular experiment, we used 4 different channels: DAPI, Far-red, red, and green. However, it's important to note that the GFP expressed in neural crest cells is cytoplasmic, whereas the GFP expressed in placode cells from the dual sensor plasmid is nuclear (nGFP). Additionally, placode cells, as opposed to neural crest cells, express nuclear RFP (nRFP) originating from the dual sensor vector. This clear distinction in subcellular localization allows us to easily differentiate between neural crest cells and placode cells.

6. For analyzing the EGFP/RFP intensity for individual placode cell, EGFP + tuj1 /RFP should be calculated to determine how many placode cells are experiencing miR-203 inhibition.

We only analyzed the EGFP/RFP intensity on placode cells that reached the ganglia (Tuj1+). Values plotted in Fig 6D represent individual cells, so it can be seen from there how many placode cells are experiencing the miR-203 inhibition.

7. For Fig. 6C, images of control experiments are required for proper comparison and analysis of the time course for GFP degradation.

Great suggestion. We have now included a time course demonstrating the lack of GFP degradation on control experiments (**Fig. S5B**).

Fig. 7:

1. Adequate information about NTA is not provided in the materials and methods section.

The M&M section has been modified for a better understanding.

Reviewer #2: This manuscript suggests a novel mechanism of cell-cell communication between neural crest and placode cells. The following comments/questions may help increase the strength and clarity of the manuscript.

We wish to convey our gratitude for the positive and constructive comments received. In response, we have made efforts to address the concerns raised by the reviewer in the points outlined below.

1. Including an insert and a zoomed in image in Figure 1 panels B and C may help readers visualize when miR-203 is present vs. absent. Also, this figure only shows a marker for neural crest cells - could a marker for placode cells also be included?

Great suggestion. We have now included higher magnification images with immunostaining for Tuj1, HNK1.

2. It remains unclear (e.g. Figure 1) in which cells miR-203 is being expressed during trigeminal ganglion formation. Is it neural crest, placodal cells or some other derivative of either of those cells?

From our new figure 1 we can observe that miR-203 at the coalescence (HH16) stage is detected in both NC and placode cells (see arrowhead in Fig. 1C'). At the condensation stage (HH20) miR-203 is mostly detected at the core of the ganglia where placode cells are mostly detected (Tuj1+).

3. The authors claim that overexpression of miR-203 generates ectopic aggregation of NC cells in figure 2. This appears to contradict their previous paper from 2019 where they claim overexpression inhibits delamination and accumulation of NC cells? Can the authors clarify? It might be important to use the previous approach from 2019 as a positive control in the explanation and findings in figure 2.

The reviewer has pointed out a very interesting observation that we had in consideration before we designed our experiments. In our previous publication, we used *ex ovo* electroporation to introduce our overexpression construct at the gastrula stage (HH5) in order to affect the early steps of NC delamination. In this new work, we used *in ovo* electroporation at HH9 embryos (5 somites), which is ~2hs before the NC starts to delaminate (6 somites). It is well documented that the promoter we use in our plasmids (chicken b-actin) usually takes ~4-5hs to be transcribed, so we will start to have miR203 overexpression way after the NC cells delaminate from the neural tube. This is the reason why we are not affecting the delamination by using this experimental design.

4. It is reported that excess miR-203 causes ectopic aggregation of neural crest cells - did the authors observe any precocious aggregation or any other effect on the timing of trigeminal ganglia formation?

This is a very interesting question, indeed we observed that the trigeminal ganglia are also bigger. However, we didn't analyze if there is an effect on the timing or if any other structure derived from NC has been affected since many of them are not reaching the correct place. But this could be an interesting point to evaluate in our future work. Thanks for the suggestion.

5. In figure 2A, can the authors use a reporter system to show that NCCs that overexpress miR-203 are the ones that don't migrate and aggregate.

We have to mention that most of the endogenous fluorescence is quenched after the ISH protocol. So, we initially visualized the embryos and photographed them before the ISH to assess for proper electroporation (those images have been included as insets in Figure 2A). Sometime after the whole mount ISH and embryo sectioning, we could still see a weak GFP signal by overexposing the sections, but this makes a lot of noise. We have now included an inset image in Figure 2A' showing the GFP channel for the corresponding section, but it is very difficult to visualize individual cells.

6. The measurement of "affected condensation" in figure 2B should probably include more description or quantification. How are defects in migration being evaluated in figure 2 in relation to condensation?

We agree with the reviewer that having a quantitation would be the best. However, this is very difficult since sometimes we observed ectopic condensation, but the ganglia were apparently normal, and some other we observed bigger/denser ganglia with just a few ectopic groups of cells. Based on this, we decided to count the number of embryos that have ectopic condensation (white arrowhead in fig 2A') and/or apparent bigger/denser ganglia (black arrowhead in fig 2A') as affected embryos. We have now included a better description of how we catalogued our embryos based on affected vs normal condensation.

7. Similarly, the authors report that four control embryos have affected condensation. Is this something that happens spontaneously in wild-type animals but is exacerbated with miR-203 overexpression? Providing more details on how images are quantified and the criteria for condensation to be categorized as "affected" would be helpful to the reader.

This is a good point. It is important to mention that in none of the cases we observed ectopic condensations in control embryos. Our control embryos presenting affected condensation are due to the observation of an uneven shape of the ganglia (bigger/denser ganglia) when comparing control versus treated sides. This could be a consequence of the manipulation of the treated side overexpressing an empty vector.

8. In experiments using the miR-203 sponge, how was loss of miR-203 function verified?

Our sponge vector was functionality validated in our previous publication (Sanchez-Vasquez et al., 2019) by indirectly measuring the miR-203 target and by doing rescue experiments. Additionally, we have now included a stem-loop RT-qPCR analysis, providing direct evidences that this sponge effectively reduces the expression of miR-203 in comparison to the scramble sponge **(Fig. S1A).**

9. In figure 3, how is targeting of placodal ectoderm validated? Are any neural crest targeted?

Our electroporation system has been a matter of concern for most of the reviewers in this manuscript and would like to clarify that this is one of the most powerful tools we have in chick embryology and it is widely used in our field. There are tons of publications demonstrating that if you electroporate the neural tube, most of the delaminating neural crest will be affected in only one side of the embryos, but none of the placode cells that have a different embryonic origin. In a similar way, we could specifically target one side of placode cells without affecting the NCC population. We have now included a better scheme of our injection and electroporation setups **(Fig. S2A-B)**. Additionally, we presented results demonstrating that transfection of neural crest with a GFP construct results in no co-localization with Tuj1 (placode marker) but clearly co-localize with HNK1 (neural crest marker) **(Fig. S2A')**. Conversely, when placode cells are transfected with a GFP construct, there is no co-localization with HNK1, but they clearly do with Tuj1 marker **(Fig. S2B')**. We hope that this experiment clarifies the specificity and robustness of our electroporation system.

In case you need further clarification, a comprehensive and detailed description of the methodology for in ovo electroporation in chick embryos to target specific tissues during early embryogenesis, can be found in Chapter 6 of the book "Neural Crest Cells: Methods and Protocols" authored by McLennan and Kulesa, which is part of the "Methods in Molecular Biology" series (Volume 1976). You can access the full chapter at this DOI link: https://doi.org/10.1007/978-1-4939-9412-0_6.

10. In figure 3b, can the authors quantify the placode condensation area for significance instead of showing significance in terms of the number of embryos with phenotype? Knocking down miR-203 didn't result in much condensation phenotype. Is this variability in terms of the size of the embryos or somite numbers.

Great suggestion. We have now quantified the trigeminal ganglia area after in situ hybridization (ISH) for Sox10 on the treated and control sides of each embryo. The analysis was performed using whole-mount images and was processed using FIJI (see Materials and Methods). The results revealed a significant reduction in the trigeminal ganglia area on the miR-203 sponge treated side, specifically when targeting placodal cells and not neural crest cells **(refer to the new scatter plots in Figure 3)**.

11. A major conclusion of this manuscript is that loss of miR-203 function in the placode cells, but not neural crest cells, leads to perturbation of trigeminal ganglia formation. If neural crest cells are the donor cells producing miR-203 that is affecting the recipient placode cells, could the authors comment on how blocking miR-203 in neural crest does not affect the production of miR-203 and subsequent sorting into sEVs? In particular, this finding undermines the overarching model presented in the manuscript.

This is a compelling observation. The speed at which the process of miR-203 biogenesis and its selective sorting into small extracellular vesicles occurs remains unknown. It is conceivable that, upon the processing of pre-miR-203 to form mature miR-203, specific RNA-binding proteins rapidly facilitate its sorting into EVs. This potential process might occur before the mature miRNA is incorporated into

the RNA-induced silencing complex (RISC), which could explain why the sponge in neural crest cells may fails to impact its functionality in recipient placode cells. This explanation is speculative, and further experiments are required to elucidate the mechanism by which miR-203 is loaded into EVs.

12. A general concern for figure 4 is that exosome secretion appears to be a rather ubiquitous phenomenon across biology, thus data supporting exosome release in trigeminal ganglia is nice, but relatively unsurprising. Related to this point, have the authors evaluated whether placodal cells secrete exosomes to NC cells?

The reviewer raised a valid point. Although it could be also very interesting, in this work we haven't evaluated if placode cells can produce vesicles that can reach the NC cells.

13. Can the authors disrupt the formation of extracellular vesicles in NCCs and show that it leads to abnormal ganglia condensation? The authors could perhaps utilize the explant culture system in transwell assay system to disrupt EVs released from NCCs and observe the effect on placode cells.

Thanks for the suggestion. Unfortunately, the explants were grown in 2D conditions and they don't have the ability to condense and form a 3D structure like the ganglia. We have tried to avoid as much as possible the use of *ex vivo* systems to really represent the most physiological condition of the NC and placode interactions.

14. Is there a method to ensure that constructs are electroporated into the correct target cells? For example, in Figure 6 the use of an additional NC marker could verify that cells electroporated with the EGFP labeled construct are indeed neural crest.

As we mentioned before, our model system allows us to specifically target each group of cells (see new **Fig. S2**).

Unfortunately, we don't have more available channels in our microscope to detect NC (we used DAPI, Far-red, red, and green). However, the GFP expressed in NC is cytoplasmic and the one expressed in placode cells is nuclear. Moreover, the NC doesn't have nRFP, so we could easily distinguish which ones are NC and which ones are placode.

15. The authors utilized an artificial system consisting of an EGFP sensor vector with miR-203 recognition sites in the 3'UTR as a read out for miR-203 function in placode cells.

This is a critical point to the manuscript. To substantiate this, can the authors sort placode cells with and without miR-203 overexpression in crest cells and assay for the following?

a) small RNA sequencing or qPCR to show increased miR-203 in placode cells?

b) mRNA sequencing or qPCR to show that predicted miR-203 targets are downregulated in placode cells?

c) Can the authors determine how downregulated miR-203 targets are involved in placode fate specification?

While we find the suggested experiments intriguing, we face a limitation in selectively sorting placode cells, preventing us from conducting the proposed experiments. Regrettably, the lack of information about the targets of miR-203 in placode cells hinders us from pursuing more direct approaches such as ISH, IHC, or qPCR.

16. The manuscript only describes a condensation defect in trigeminal ganglia. How does perturbation of miR-203 in crest or placode cells affect further maturation or function of trigeminal ganglia?

Assessing the functionality of the trigeminal ganglion in embryos with disrupted miR-203 would be highly intriguing. Unfortunately, we lack the capability to conduct such studies in chickens.

17. The experimental paradigm for figure 6 is novel and complex, further validation of the system would strengthen the interpretation of the results.

We value the reviewer's acknowledgment of the complexity of this challenging experiment and acknowledge the concern for additional validation. Therefore, recognizing the limitations of the experimental model employed, we have substantiated the reproducibility of our findings through both in vivo and ex vivo approaches.

18. In figure 7, the notion of selective loading is poorly supported. Further controls are needed. For example, dissected TG samples is a heterogenous mix of cells and presumably the vesicles are a heterogeneous mixture from different cell types. Selective sorting of miRNAs into exosomes is only

beginning to be understood and this manuscript is not focused on dissecting this phenomenon, thus figure 7 is trying to tackle a much bigger question in the field.

We totally agree with the reviewer and have softened our conclusions.

19. Some of the movies shown in this manuscript are not accompanied by quantification or report of how many replicates were performed. Adding this information would strengthen the conclusions drawn from these results and provide context for readers.

Thanks for pointing this out. We have now included the number of replicates performed on each experiment. There were 6 replicates for the co-cultures pHluo (NC) + mRFP (Placode), 6 replicates for OVE miR-203 (NC) + dual sensor (Placode), and 6 replicates for empty vector CTRL (NC) + dual sensor (Placode).

To summarize, the interaction between NC and placodal cells is an understudied aspect of development and the idea of signaling by exosomes is quite interesting, however, the data in the manuscript mildly supports this mechanism and leaves this reviewer more interested in how miR-203 regulates mRNA transcripts to affect cell behavior. The current version of the manuscript doesn't address how miR-203 affects condensation of trigeminal ganglia.

We firmly believe that our discovery, although we have yet to identify the direct targets of this miRNA in placode cells, represents a novel contribution. For the first time, we have demonstrated the essential role of miR-203, originating in the neural crest, being sorted into extracellular vesicles, and playing a crucial role in placode cells during trigeminal ganglion development. Moreover, it is very interesting how this miRNA is epigenetically silenced during the initiation of the NC EMT (our previous work, Sánchez-Vásquez et al., *Dev* 2019), and we have now demonstrated the re-activation in a subset of NC during their coalescence with placode cells. Performing such experiments in an in vivo system is undeniably challenging, and we hope that you find the revised version of the manuscript, which includes substantial new information and validation, compelling enough for further publication.

Reviewer #3: This is excellent work that is extremely challenging to perform in vivo in a non-model organism and may benefit from some of the comments and requests for controls listed below. The contribution presents outstanding evidence that miR-203, and EVs have in embryonic development.
We want to express our gratitude to the very positive and constructive comments. We have tried to address most of the reviewer concerns in the following point.

Figure 2. Which cells received the plasmids through electroporation? Since the promoter is not specific to a cell type, it is unclear whether non-target cells receive miR-203. The authors should confirm that miR-203 is expressed in ectopically positioned cells through co-electroporation of additional plasmids.
Our electroporation system has been a matter of concern for most of the reviewers in this manuscript and would like to clarify that this is one of the most powerful tools we have in chick embryology and it is widely used in our field. There are tons of publications demonstrating that when you electroporate the neural tube most of the delaminating neural crest will be affected in only one side of the embryos, but none of the placode cells that have a different embryonic origin. In a similar way, we could specifically target one side of placode cells without affecting the NCC population. We have now included a better scheme of our injection and electroporation setups **(Fig. S2A-B)**. Additionally, we presented results demonstrating that transfection of neural crest with a GFP construct results in no co-localization with Tuj1 (placode marker) but clearly co-localize with HNK1 (neural crest marker) **(Fig. S2A')**. Conversely, when placode cells are transfected with a GFP construct, there is no co-localization with HNK1, but they clearly do with Tuj1 marker **(Fig. S2B')**. We hope that this experiment clarifies the specificity and robustness of our electroporation system.
For a comprehensive and detailed description of the methodology for in ovo electroporation in chick embryos to target specific tissues during early embryogenesis, we refer you to Chapter 6 of the book "Neural Crest Cells: Methods and Protocols" authored by McLennan and Kulesa, which is part of the "Methods in Molecular Biology" series (Volume 1976). You can access the full chapter at this DOI link: https://doi.org/10.1007/978-1-4939-9412-0_6.
Figure 3. Similarly, the authors use a "sponge" that is electroporated to sop up miR-203 and compete with potential target mRNA sequences. However, no control experiments confirm the expression of the sponge in the target cells.
The reviewer raised a valid observation. Our sponge vector was functionality validated in our previous publication (Sanchez-Vasquez et al., 2019) by indirectly measuring the miR-203 target and by doing rescue experiments. We have now included a stem-loop RT-qPCR analysis, providing direct evidences that this sponge effectively reduces the expression of miR-203 in comparison to the scramble sponge **(Fig. S1A).**
Figure 4. The approximate localization of pHluo puncta to Tuj1 positive neurons likely means that the EVs are attached to the outside of neurons since the authors argue that the fluorescence is PH dependent. It would be interesting then to stain for the fluorescent protein to determine whether any is endocytosed. In addition, are the authors proposing that the miRNA escapes from the endocytosed EVs or that the EVs fuse with the cell membrane?
This is a very interesting point. We still don't know how the EVs are endocytosed by placode cells, but not all the methods involve an acidic compartmentalization that can turn off pHluo. Moreover, it is possible that the time used in our time-lapse co-cultures or the *in vivo* experiments were not sufficient to see the turn-off of the pHluo protein inside the placode cells. We have shown some Z-stacks and 3D rotation that show that neural crest produced EVs that are internalized by placode cells. We speculated that EVs could be captured by filopodia or macropinocytosis events.
Figure 4A requires further explanation. In addition, in F-G', it would be useful to have a scale bar.
We have now included a better description of the figure in the mail text and the scale bars in all our micrographs.
Figure 5. The distribution of CD63 is eerily similar to migrasomes and commentary regarding this similarity might be warranted.

We totally agree with the reviewer that this pHluo construct produced cell-trails that highly resemble migrasomes and retraction fibers. Indeed, a similar structure have been also described in the original work utilizing the same pHluorin-CD63 vector (sung et al., 2020). Migrasomes is highly correlated with the migration of cells, and neural crest may not be an exception. Currently, it has been demonstrated the migrasomes may also been involved in lateral transfer of mRNA or proteins acting as developmental cues in embryos to modulate organ morphogenesis (Jiang et al., 2019). Thus, migrasomes have been proposed to provide a mechanism for integrating and relaying spatiotemporal chemical information for cell–cell communication, maybe mediated by miRNAs, and this is something that we are now studying in the lab in the context of neural crest migration.

Figure 6B and B' have a strange line near the center of my image. It appears that the image may be a composite. If this is true, the authors might wish to separate this or to discuss this. It could be an issue with my PDF.

In Figure 6B we used an automatic tiles imaging tool provided by ZEN program to be able to visualize the entire trigeminal ganglion and the electroporated placode cells with the sensor vector. This is why you see that line where the program automatically joins the images. However, in the case of figure 6B', this is a single image and it shouldn't have any line. We will also include individual TIFF images for a better resolution in case that it is an issue with your PDF.

Figure 7. The authors indicate that the sEVs are from trigeminal ganglia, but is there a reason that the data are not from neural crest cells along?

The reviewer appreciation is correct. We fully concur with the reviewer's observation that we likely have a heterogeneous mix of sEVs originating from both neural crest and placode cells. As we need to obtain sufficient material for sEV isolation and subsequent extraction of their RNA content for stem-loop RT-PCR, we isolated small extracellular vesicles (sEVs) from approximately 100 dissected trigeminal ganglia for each experiment done. The process of dissecting such a significant number of ganglia is time-consuming. We acknowledge that sorting cells derived from neural crest to isolate a pure population of sEVs would have been ideal but is technically impossible within the context of in vivo condensation.

General Comments.

A. Can the authors please provide further methodological details regarding electroporating the two distinct cell populations. This is a wonderful set of experiments/techniques and it would be useful to further elaborate in methods for each set of experiment the different conditions.

Thank you for bringing this to our attention. We have taken steps to incorporate electroporation schemes into most of our figures. Furthermore, we have created a new supplementary figure S2, which provides a concise overview of the methodological aspects related to electroporating the two distinct cell populations. This supplementary figure also includes illustrative images and immunostaining that vividly demonstrate the effectiveness of our system, a widely recognized and utilized approach in our field. For a comprehensive and detailed description of the methodology for *in ovo* electroporation in chick embryos to target specific tissues during early embryogenesis, we refer you to Chapter 6 of the book "Neural Crest Cells: Methods and Protocols" authored by McLennan and Kulesa, which is part of the "Methods in Molecular Biology" series (Volume 1976). You can access the full chapter at this DOI link: https://doi.org/10.1007/978-1-4939-9412-0_6.

The authors propose that they are electroporating miR-203 into a single cell population, but there is no data to demonstrate this. Moreover, if miR-203 from the plasmid is released, it could have widespread effects, but the authors do not indicate where the specificity of the effect is coming from. Is the specificity of the endogenous or ectopic expression experiment derived from the specific targeting of the EVs, the miRNA, or the target inside of placode cells?

This is an excellent comment that make us to think about how a subset of NC has the ability to exclusively communicate with PC during ganglion assembly. In this regards, it is known that ablation of cranial NC subgroup or their replacement by trunk NC causes abnormalities in trigeminal ganglion assembly, mostly by impacting the integration and differentiation of PC into the ganglion. This

highlighted the idea that those cranial NC has the intrinsic ability, maybe because they are able to reactive miR-203 expression, to interact with PCs. In our overexpression experiments, we are impacting the migratory neural crest (NC) from various axial levels. The elevated levels of miR-203, along with the potential over-release into small extracellular vesicles, might also influence other mesenchymal cells, leading to their coalescence and the formation of the observed ectopic aggregates of neural crest cells.

Have the authors considered transplanting EVs containing over-expressed miR-203 or EVs from cells electroporated/transfected with the sponge so that the EVs are devoid of miR-203?
This is a very interesting experiment, but we have to mentioned that getting enough electroporated embryos to dissect the ganglia and then purify enough sEVs for doing the proposed experiments would be technically very challenging.

Are there additional miRNA or proteins in EVs that may be causing the effects seen?
We posit that other microRNAs might be functioning similarly to our proposed mechanism for miR-203. We believe there could be a core set of microRNAs specifically sorted into small extracellular vesicles that play a role in the intercellular communication between neural crest and placode cells. Currently, we are engaged in miRNA sequencing and proteomic analysis of sEVs during the formation of the trigeminal ganglion. While data analysis is ongoing, we are also initiating the functional validation of certain RNA-binding proteins that could potentially serve as miRNA sorting proteins. We look forward to presenting these results in our upcoming article.

Reviewer #4: While the data presented are novel in the sense that they provide a communication mechanism between NCCs and placodal cells, they do not investigate the targets within placode cells to provide more mechanistic insight. It is also not clear why placode cells specifically take up EVs while other cells that NCCs encounter on their migratory route do not. Indeed in Fig. 4D there are many other non-trigeminal cells that take up EVs. So, one is left wondering how this process works mechanistically and how specificity is achieved.
Nevertheless, the experiments nicely combine in vivo and in vitro experiments thus putting their findings into a biological context. There are several points the authors should address beyond providing mechanistic insight.
We greatly value the constructive feedback provided by the reviewer, and we have taken diligent efforts to address the majority of the concerns raised in the subsequent points. As the reviewer mentioned the major goal of this work was to demonstrate the *in vivo* mechanism between NCC and placode cells required for the aggregation. We concur with the reviewer's observation that we have yet to identify the specific targets of miR-203 within placode cells. We wish to emphasize that we are actively engaged in the development of specialized tools that will enable us to selectively label and sort placode cells, a process that regrettably necessitates a considerable amount of time. We do not assert that placode cells are the only one able to take up EVs, but perhaps they are the ones capable of responding or changing their phenotype after their uptake due to the genes they express which may or may not be targeted by specific miRNAs contained into EVs.
We acknowledge the reviewer's observation that comprehending the intricacies of cell-to-cell communication through EVs, particularly within the intricate milieu of *in vivo* systems, remains an evolving field. Factors such as neighboring tissues and the presence of extracellular matrix components certainly contribute to the complexity of this phenomenon. Nevertheless, we remain confident that our work offers a substantial contribution to this burgeoning area of research, offering novel insights and a mechanistic framework. We believe that our study has the potential to significantly advance our understanding of the underlying processes at play in the context of NCC-placode cell interactions and cell-to-cell communication via EVs within *in vivo* systems.

Fig. 2. These experiments should include a lineage tracer in addition to the overexpression constructs. This will allow the authors to assess whether the ectopic aggregates are derived from the cells containing the construct or whether other, non-neural crest cells are recruited. This is particularly important because the authors later show a non-cell autonomous effect.
We thank the reviewer for pointing this out. We have to mention that most of the endogenous fluorescence is quenched after the ISH protocol. So, we initially visualized the embryos and photographed them before the ISH to assess for proper electroporation (those images have been included as insets in Figure 2A. Sometime after the whole mount ISH and embryo sectioning, we could still see a weak GFP signal by overexposing the sections, but this makes a lot of noise. We have now included an inset image in Figure 2A' showing the GFP channel for the corresponding section.
Our electroporation system has been a matter of concern for most of the reviewers in this manuscript and would like to clarify that this is one of the most powerful tools we have in chick embryology and it is widely used in our field. There are tons of publications demonstrating that when you electroporate the neural tube most of the delaminating neural crest will be affected in only one side of the embryos, but none of the placode cells that have a different embryonic origin. In a similar way, we could specifically target one side of placode cells without affecting the NCC population. We have now included a better scheme of our injection and electroporation setups **(Fig. S2A-B)**. Additionally, we presented results demonstrating that transfection of neural crest with a GFP construct results in no co-localization with the placode marker (Tuj1), but clearly co-localize with the neural crest marker (HNK1) **(Fig. S2A')**. Conversely, when placode cells are transfected with a GFP construct, there is no co-localization with HNK1, but they clearly do with Tuj1 marker **(Fig. S2B')**. We hope that this experiment clarifies the specificity and robustness of our electroporation system.

For a comprehensive and detailed description of the methodology for in ovo electroporation in chick embryos to target specific tissues during early embryogenesis, we refer you to Chapter 6 of the book "Neural Crest Cells: Methods and Protocols" authored by McLennan and Kulesa, which is part of the "Methods in Molecular Biology" series (Volume 1976). You can access the full chapter at this DOI link: https://doi.org/10.1007/978-1-4939-9412-0_6.

2B: provide a better label for 'affected' condensation; this could mean more or less - be specific. Use 'enhanced', 'increased' or similar.

The reviewer raised a valid point. In our phenotypes sometimes we observed ectopic condensation but the ganglia were apparently normal, and some other we observed bigger/denser ganglia with just a few ectopic groups of cells. This is why we decided to count the number of embryos that have ectopic condensation (white arrowhead in fig 2A') and/or apparent bigger/denser ganglia (black arrowhead in fig 2A') as named them as "affected embryos". We have now included a better description of how we catalogued our embryos based on affected (ectopic and/or enhanced condensation) vs normal condensation.

Fig. 3: maintain colour scheme for NC or placode cells introduced in Fig. 1, except for colour coding electroporating cells in green.

Given our modification of the color scheme in Figure 1 to illustrate the co-localization of miR-203 expressing cells with placode (Tuj1+ in green) and NC (HNK1+ in red), we have opted to maintain this color code in Figure 3. This decision is intended to provide clarity, helping to exemplify the specific cells transfected through electroporation.

3B: scrambled and miR-203 sponge electroporated embryos should be the same stage. As above, including of lineage tracer in these experiments is crucial to appreciate whether the effect is observed in electroporated cells or in their neighbours. The effects are difficult to see and more convincing images are required.

It is worth noting that although we meticulously selected embryos at identical developmental stages, inherent variability does exist, leading us to consistently present both the control and treated sides for each individual embryo. In addition to this, we have introduced a quantitative assessment of the trigeminal ganglia area (see scatter plots in Fig. 3) in order to provide a more precise representation of the impact of each treatment on the ganglia. This addition is aimed at offering a clearer understanding of the alterations observed within the trigeminal ganglia in response to the treatments.

The authors should use their explant experiment to show that overexpression and inhibition of miR-203 causes changes in cell behaviour in placode cells. Explants would allow better quantification and imaging cells over time, and provide more solid data.

We appreciate your valuable suggestion. Regrettably, the explants under investigation were cultivated under 2D conditions and, as a consequence, lacked the inherent capability to undergo condensation and form a 3D structure akin to ganglia. Our primary endeavor has been to minimize the utilization of *ex vivo* systems, in order to faithfully replicate the most physiologically relevant conditions for studying interactions between NC and placode cells.

The statement in line 262/263 that effects are more severe in ophthalmic thank in the maxilla-mandibular lobe is not justified by the results presented. First this may be a result of uneven electroporation (again lineage tracer will be essential to distinguish); second no data are provided to support this statement. Finally, the different branches and their significance were not introduced anywhere, so only a specialised reader can appreciate this fact.

Thanks for the pointing this out. We have now removed this sentence from the text.

Fig. 4 A: explain colour code of cells. B, C: provide better labels to guide the reader e.g. where is the plasma membrane, extracellular space etc.

Thanks for the suggestion. The colors in figure 4A is just showing different cells at the condensing ganglia contacting each other. We have now included labels localizing the extracellular space (ExS) and plasma membrane (PM) in B and C.

In panels D-G the authors aim to show that NC secrete EVs that are then taken up by placode derived trigeminal neurons. As presented the data are not entirely convincing. The authors state that cells in F are neural crest cells; there are no markers to show that they are. How do the authors exclude that they are other cell types?

In line with our previous description, our electroporation system demonstrates a high degree of specificity in its targeting capabilities, enabling us to confidently assert our ability to exclusively target the neural tube with the pHluo marker. Leveraging this precision, we can confidently deduce that all the migratory cells observed within the mesenchyme originate from the neural crest, as these are the sole cell population known to undergo delamination and subsequent migration from the neural tube. To provide comprehensive support for our methodological approach, we have thoughtfully included a new supplementary figure (**Fig. S2**), which serves as an exemplar of our experimental methodology. Furthermore, we have enriched our findings with a co-immunostaining assay employing HNK-1, a marker indicative of neural crest cells, as depicted in **Figure S3A**. This co-immunostaining unequivocally demonstrates that the migratory pHluo-positive cells are, indeed, neural crest cells, reinforcing the robustness and accuracy of our experimental outcomes.

In G/G' the authors state that placode derived neurons contain EVs. To identify these neurons they use Tuj1, which labels axons - it is difficult to appreciate that their cell bodies contain EVs. It would be better to include also membrane marker.

Anti-Tuj1 uniquely label placodal cells during early ganglion assembly and have been used in several publications in chick embryos demonstrating to have a beautiful staining in cells bodies too (Shiau et al., 2009 PMID: 19934013; and Shiau et al., 2008 PMID: 18278043).

The explant experiments are much clearer showing that EVs are transferred from NCs to placode cells. However, how can the authors be sure that these are cytonemes? It might be better to be more careful in their use of terminology.

We thank the reviewer to the appreciation of our experiment. We totally agree and will soften the use of our terminology and describe them as cytonemes-like structures.

Minor comments.

It would be good to cite more original papers than reviews. For example:

Line 66: cite original paper, not review Line 69: cite original paper, not review Line 231 'ectopic aggregation', not 'ectopically'.

All the changes have been made accordingly to the reviewer suggestion.

The scheme in Fig.1 A suggests that placode derived cells locate to the centre of the TG and NC derived cells to the periphery; if this is true, please cite the evidence.

Yes, cranial neural crest cells form corridors prefiguring placodal cell migration. This has been published by Freter et al (PMID: 23942515). We included this in the references.
