# Supplementary material for: miR-203 secreted in extracellular vesicles mediates the communication between neural crest and placode cells required for trigeminal ganglia formation
Source: PLoS Biol. 2024 Jul 22;22(7):e3002074. doi: 10.1371/journal.pbio.3002074 (PMC11293684; doi:10.1371/journal.pbio.3002074)
Supplement: Attachment — Submitted filename: REVIEWS-R2.pdf [file pbio.3002074.s016.pdf]

Reviewer #1: The authors have addressed most of the technical concerns raised. They have carried out additional experiments which lend further support to the conclusions drawn. The revised manuscript may now be considered as suitable for publication.

Reviewer #2: We thank the authors for addressing our concerns by including additional data and analysis, supplementary figures, and resources for our understanding. We feel that this manuscript addresses an interesting mechanism involving miRNA within exosomes that communicate between two distinct cell-types as they converge to generate placode tissue. While the data here support the importance of miR-203 in trigeminal ganglion cells, the central conclusion that NC cells secrete EVs loaded with miR-203 to communicate with placode cells remains poorly supported. The authors do present data that miR-203 overexpression in NC cells represses a miR-203 sensor in placode cells, and that EVs from the trigeminal ganglion contain miR-203; however, they fail to show that miR-203 is not expressed in placode cells which could confound their conclusion. Additionally, the authors include data suggesting that blocking miR-203 in NC cells has no effect on trigeminal ganglion condensation which challenges the conclusion that this miRNA is important for NC cell and placode communication during this developmental period. The authors cite limitations that prevent (1) sorting of placode cells and (2) inhibition of EV formation/secretion (e.g. genetic or pharmacological), both of which would significantly improve the support for the conclusions made in this manuscript. To conclude, the idea that two distinct lineages of cells communicate via exosomes to control tissue/organ formation is provocative, thus our critical focus on this point.

We greatly value the feedback provided by the reviewer and acknowledge the concerns regarding the origin of miR-203. In response, we conducted a new experimental approach targeting the genomic locus of miR-203 in either neural crest or placode cells using CRISPR-Cas9 technology.

Initially, we demonstrated that the electroporation of two vectors containing specific gRNAs, situated at the miR-203 locus, along with a Cas9-containing vector, resulted in the generation of INDELS, as evidenced by HMA PCR, and a reduction in miR-203 expression, as shown by stem-loop RT-qPCR (refer to new Fig. S1B). Embryos were also electroporated with a control vector containing a scramble gRNA for comparison.

Our previous experiments utilizing a sponge vector indicated the necessity of miR-203 in placode cells, but not in neural crest cells, for proper ganglion condensation (refer to Fig. 3A-B). However, our latest findings using CRISPR-Cas9 reveal that trigeminal ganglion condensation is affected only when specific gRNAs and Cas9 are electroporated into neural crest cells, not placode cells (see new Fig. 3C-D). This unequivocally demonstrates that neural crest cells are the source of miR-203 transcription, while placode cells are its sites of action.

We hypothesize that the sponge vector may not efficiently capture the produced miR-203 in neural crest cells, possibly because the miR-203 is not yet loaded into the RISC complex or rapid sorting into small extracellular vesicles. However, further experimentation is required to confirm this assumption.

We trust that these new findings address the concerns raised by the reviewer and significantly enhance the robustness of our study. We sincerely appreciate the constructive comments provided, which have greatly contributed to the advancement of our research.

Reviewer #3, David M. Feliciano (note, Reviewer 3 has signed this review): The authors of the manuscript adequately answered all questions and responded to all comments. The manuscript is an excellent piece of science working on an extremely challenging set of questions in developmental biology. The experiments provide compelling evidence that EVs function as an important developmental morphogen.

Reviewer #4: The authors have addressed some points raised by the reviewers. Mostly, they toned down their conclusions or provided more explanation.

The issue of tracing precisely the electroporated cells was raised by several reviewers and remains an issue, in particular since the authors aim to provide evidence for non-cell autonomous effects. I do appreciate electroporation as a strategy for functional experiments in chick and am aware that this is used by many labs. However, it is standard in the field to include a lineage tracer. The authors have included some data to increase confidence in their electroporation strategy and argue that GFP does not survive processing of the embryos. This is easy to resolve by using antibodies for post-staining, again done by many groups in the field. My enthusiasm for the manuscript remains reduced because of the lack of mechanistic understanding and cell-specific uptake of EVs.

We value the feedback provided by the reviewer regarding the necessity of GFP staining. Despite our efforts to address this concern, it remains unclear whether GFP staining is an essential requirement. In response, we have included electroporation GFP images as insets in all micrographs, adhering to the conventional approach widely adopted in chick-related studies to showcase electroporation efficiency.

We believe that we have furnished a wealth of valuable information and alternative methodologies, including the incorporation of new figures utilizing CRISPR/Cas9 to target miR-203. These additions strongly support our hypothesis. For this reason, we do not believe that the absence of using a lineage tracer could be a contributing factor in the decision to reject our manuscript.
